# Thriving Through Relationships in Sport: The Role of the Parent–Athlete and Coach–Athlete Attachment Relationship

**DOI:** 10.3389/fpsyg.2021.694599

**Published:** 2021-08-02

**Authors:** Louise Davis, Daniel J. Brown, Rachel Arnold, Henrik Gustafsson

**Affiliations:** ^1^Department of Psychology, Umeå School of Sports Science, Umeå University, Umeå, Sweden; ^2^School of Sport, Health and Exercise Science, University of Portsmouth, Portsmouth, United Kingdom; ^3^Department for Health, University of Bath, Bath, United Kingdom; ^4^Faculty of Arts and Social Sciences, Karlstad University, Karlstad, Sweden; ^5^Department of Sport and Social Sciences, Norwegian School of Sport Sciences, Oslo, Norway

**Keywords:** attachment styles, competition, performance, well-being, parents, relationships, coaches

## Abstract

The aim of this research was to examine whether attachment relationships to significant others, such as to parents and/or sports coaches, enable thriving and competition performance within sport. Two studies employing cross-sectional and prospective designs were carried out across different samples of athletes of varied skill levels and sports. In Study 1, we found athletes’ attachment to their sports coach was significantly associated with athlete thriving and mediated by psychological needs satisfaction. Results of Study 2 found that athletes’ secure attachment to their mother and/or father positively predicted the experience of thriving at the competition while athletes’ insecure attachment did not predict thriving. Furthermore, athletes’ attachment to both mother and father did not predict competition performance. Together, these two studies acknowledge the significant role that athletes’ secure attachment relationships with parents and coaches play in facilitating thriving in athletes. These findings have significant implications for research and practice.

## Introduction

Sport performers encounter a variety of stressors, hassles, and adversities as part of their involvement in competitive sport, with responses to such demands having powerful effects not only on sporting performances but also on athletic well-being (Jones and Hardy, 1990; Arnold and Fletcher, 2021). Despite academic literature seeking to examine, understand, and promote *both* performance and well-being, recent media coverage indicates that an unrelenting need to succeed within the realms of elite sport can create detrimental and harmful environments where performance and results are given priority at the expense of athletic welfare (Grey-Thompson, 2017; Phelps et al., 2017; Brown et al., 2021b; Kavanagh et al., 2021). This focus also appears to be evident in youth sport, with reports illustrating concerning numbers of young people experiencing emotional harm or child abuse while taking part in sport (Hartill and Lang, 2018). Therefore, a pressing and important issue in contemporary sport is how performance can be enhanced while simultaneously optimizing well-being within highly demanding environments.

In support of the growing calls to protect athlete well-being in the pursuit of performance (Arnold and Fletcher, 2021) and the subsequent re-stating and development of welfare policies (Kavanagh et al., 2021), scholars have begun to pursue an agenda toward the promotion of thriving in sport (Brown et al., 2021b). *Thriving* describes the concurrent perception of a high-level of performance and experience of high levels of well-being within a specific sporting encounter (e.g., a match; Brown et al., 2020a) or an overall perception of high levels on both dimensions over a sustained period (e.g., a month; Brown et al., 2017b; see also, Brown et al., 2018). Given the subjective nature of perceptions and experiences, the occurrence of thriving is understood from the viewpoint of an individual evaluating one’s own functioning (e.g., do I perceive that I performed at a high-level in today’s match?). As such, the construct of thriving has been qualitatively explored via the lived experiences of individuals operating in sport (see, e.g., Brown and Arnold, 2019) and quantitatively identified via their self-reported accounts on performance and well-being dimensions (see, e.g., Brown et al., 2017b; McNeill et al., 2018). When researching thriving in sport, it has been important for scholars to recognize the full and holistic nature of thriving (see, Brown et al., 2017a; Ryan and Deci, 2017), whereby the participants would be expected to demonstrate high levels across multiple, context-relevant functioning indicators to be labeled as thriving (Brown et al., 2020b). Quantitatively, this has been evidenced through the work of Brown et al. (2017b) who conducted factor mixture analysis to determine the shape and level of functioning profiles with a sample of 535 sport performers. Their results demonstrated no shape effects with performers reporting comparable perceptions on subjective performance, eudaimonic well-being, and hedonic well-being measures, ranging from high (i.e., thriving) to low levels. When combined with the wider evidence from McNeill et al. (2018), Brown et al. (2020a), and Rouquette et al. (2021), these findings suggest that proxies for functioning can be modeled with a single, global factor (i.e., functioning/thriving).

Within the initial work on thriving, researchers have identified various psychosocial variables associated with its occurrence. Adopting the categorization offered by Brown et al. (2017a), these variables can be broadly categorized as personal (i.e., individual attitudes, cognitions, and behaviors) and contextual (i.e., environmental characteristics and social agents) enablers. Examples of personal enablers of thriving in sport have included desire and motivation, goal setting and creating challenge, positive mental state, self-belief, mental toughness, self-regulation, and personal resilient qualities (Brown et al., 2017b, 2018; Gucciardi et al., 2017; McNeill et al., 2018). Turning to contextual enablers, these have included the depth and sincerity of relationships and the support that can be provided by coaches, support staff, parents, and colleagues/teammates (Harris et al., 2012; Brown et al., 2017b; Gucciardi et al., 2017; Brown and Arnold, 2019). Further research is, however, required on the relationship between contextual enablers and thriving in sport, given that Brown et al. (2017b) contrastingly found that perceived social support, coach need support, and coach need thwart variables could not significantly predict sport performers’ membership to a thriving profile.

One contextual enabler that is of particular interest in future enquiries is a sport performer’s attachment to significant others, such as to their parents and/or coaches. Outside of sport, research has found that interpersonal relationships built on secure attachments can act as a contextual enabler for thriving across the lifespan (see, e.g., Haynes et al., 1984; Carver, 1998; Feeney and Collins, 2015a,b). Indeed, Feeney and Collins (2015a,b) present a model of thriving which, rooted in and providing advances to attachment theory (Bowlby, 1969/1982), positions relationships as central for enabling thriving through two life contexts. These are: successfully coping with adversity (by helping to strengthen *as well as* protect) and participating in opportunities for growth in the absence of adversity (with support providers serving as *active catalysts* for thriving). Given these empirical links found outside of the sports context and the aforementioned importance of promoting thriving in sport, it is critical that future research investigates attachment as a contextual enabler of athletic thriving.

The term “attachment” refers to an individual’s ongoing emotional bond with a significant figure (usually the mother or a significant caregiver) upon whom s/he has learned to rely on for protection and care (Bowlby, 1969/1982). Differences in the ability of a child to signal the need and desire for closeness, as well as differences in a caregiver’s responsiveness to the needs of their child, produce variations in what Ainsworth et al. (1978) labeled *attachment styles*. Alongside of which, a set of knowledge structures or internal working models (IWMs) are formed that are cumulative representations of the self (child) and of significant others (caregivers). Based on Bowlby’s theories, Ainsworth et al. (1978) identified three styles of child attachment: secure, anxious ambivalent, and avoidant. When a parent demonstrates availability, is sensitive to signals of distress, and responsive when called upon for protection and/or comfort, a *secure* attachment style is developed. The IWM of a secure individual includes trust in the caregiver and confidence in the availability and provision of support should the individual encounter adverse or frightening situations. With this assurance, secure individuals are generally bold in their explorations of their environments as they are able to rely on themselves and others when needed; they are also comfortable with relational closeness. An anxious ambivalent attachment style is developed when a caregiver is inconsistent in their availability, reassurance, and providing protection and/or comfort (e.g., being available and supportive on some occasions and not on others). The IWM of an anxious individual includes uncertainty as to whether the caregiver will be available, responsive, or supportive when called upon. Due to this uncertainty, an anxious individual has a lack of trust in their caregiver, a fear of rejection, and a strong need for relational closeness (Cassidy, 1994). Lastly, when a caregiver constantly rejects a child when s/he approaches for comfort and/or protection, an avoidant attachment style is developed. The IWM of an avoidant individual includes negative self-evaluations and a lack of confidence that their caregiver will be accessible and responsive when called upon. On the contrary, they expect to be rejected and the importance of caregiver availability is minimized and relational closeness is avoided (Cassidy, 1994).

Research on parent–child attachment has been conducted across a variety of domains (e.g., familial, social/friendships, education, sport; Zimmermann, 2004; Ramsdal et al., 2015) and at different phases of a lifespan (e.g., infancy, childhood, adolescence). A secure attachment is considered important for the development of positive social–emotional competence, cognitive functioning as well as good physical and mental health including well-being (Mónaco et al., 2019). In general, previous research has found those with insecure attachments to be more at risk from developing negative outcomes and ill health (Gillath et al., 2016).

In relation to the context of sport, studies that have focused on the parent–child attachment relationship have investigated links with engagement and motivation for physical activity, physical self-concept (Ullrich-French et al., 2011; Li et al., 2016) as well as the development of sporting friendships (Carr, 2009). Collectively these studies have demonstrated a strong positive link between mother and father secure attachment and motivation for physical activity as well as positive links to athletes’ physical self-perception (Ullrich-French et al., 2011; Li et al., 2016). Furthermore, Carr (2009) found that attachment to parents played a significant role in influencing how sporting friendships were formed within the context of sport. On the contrary, across all studies, attachment insecurity was notably most detrimental to these outcomes. Notwithstanding these associations, parent–athlete attachment is yet to be shown to influence sport performance and no previous studies have examined the relationship with thriving in sport.

In addition to influencing child–parent relationships, once developed, IWMs act as a prototype and play an important role in shaping close relationships and can guide the formation of future attachments including those with leaders, teachers, friends, and sports coaches (Collins and Read, 1990; Bergin and Bergin, 2009; Mayseless, 2010; Davis and Jowett, 2014). That said, across these relationships a person’s IWMs may undergo revision or be replaced when changes occur in parental caregiving (Egeland and Farber, 1984) or when a person has a corrective experience, such as the development of a supportive and sensitive relationship. Not all people interact in the same way and thus, it is possible to have working models and attachment styles that reflect the nuances connected with different relationships (Overall et al., 2003). For instance, individuals can hold a set of representations for relationships with parents, and another set of representations for their peers (Gillath et al., 2016).

In recent years, this framework has begun to examine contextual relationships in sport beyond the parent–child relationship including the coach–athlete relationship and sport friendships (Carr, 2009; Felton and Jowett, 2013; Davis and Jowett, 2014). With regard to the coach–athlete relationship, Davis and Jowett (2010) argue that coaches can take on a “stronger and wiser” role by providing support, advice, guidance, and comfort as well as encouraging exploration and risk-taking behaviors, similar to the role of parents. On this premise, Davis and Jowett (2010) found coaches to fulfil the basic functions of attachment (i.e., proximity maintenance, safe haven, secure base) essential for an attachment relationship to occur (Hazan and Shaver, 1994). Specifically, athletes reported turning to their coach during times of need, seeking a level of closeness with their coach, and relying on them to explore and discover aspects of their sporting environment. Based on this initial evidence, Jowett and colleagues investigated links between coach–athlete attachment and athlete’s affective well-being (Felton and Jowett, 2013; Davis and Jowett, 2014), sport satisfaction (Davis and Jowett, 2010, relationship quality (Davis et al., 2013), and eating psychopathology (Shanmugam et al., 2011). Findings have indicated that avoidant and anxious attachment styles are negatively linked to relationship satisfaction, sport satisfaction (i.e., satisfaction with their training and instruction, personal treatment, and performance) and well-being including vitality, and positive affect. On the contrary, when athletes reported low levels of attachment anxiety and avoidance (i.e., a secure attachment) they reported high levels of well-being (Davis and Jowett, 2014), Furthermore, this relationship has found to be most significant when all three psychological needs (e.g., autonomy, competence and relatedness) are satisfied (Felton and Jowett, 2013). Although not yet associated directly with performance, these findings suggest that coach–athlete attachment may offer an important enabler of thriving.

Within both the thriving and attachment literatures, basic psychological need satisfaction has been shown to be a key variable of interest. To elaborate, within the thriving literature, satisfaction of basic psychological needs has been forwarded as a pre-requisite and proximal determinant of thriving (see Sheldon, 2009; Mahoney et al., 2014; Brown et al., 2017a; Ryan and Deci, 2017). Indeed, Ryan and Deci (2017) suggest that humans are thought to achieve full functioning (or thriving) through the satisfaction of the basic and universal psychological needs of autonomy, competence, and relatedness. With regard to sport-based evidence, basic psychological need satisfaction has been shown to be a reliable predictor of thriving across cross-sectional (Brown et al., 2017b), longitudinal (Brown et al., 2021a), and prospective (Brown et al., 2020a) studies. Turning to the relationship between attachment and basic psychological need satisfaction, Felton and Jowett (2013, 2017) have found that basic psychological need satisfaction mediates the relationship between coach–athlete attachment and parent–athlete attachment on athlete’s well-being (vitality, positive and negative affect). Thus, when examining the possible relationship between attachment and thriving, it appears important that basic psychological need satisfaction is also considered as a potential mediating variable in this relationship.

### The Present Study

The overarching aim of this paper was to add to the small body of emerging work on athlete thriving by examining “if” and “how” relationships to significant others, such as to parents and/or sports coaches enable (or hinder) thriving within sport. While research has attempted to examine both contextual enablers (attachment relationships) and process variables (basic psychological needs) on separate indicators of thriving (specifically, well-being), research has not yet examined such enablers of thriving as it has been conceptualized within sport to include indicators of well-being and performance in tandem. Thus, this paper presents two studies. Study 1 aims to extend previous research by examining: (1) the relationship between coach–athlete attachment and thriving across a variety of sports and (2) the mediating effects of basic psychological need satisfaction on the relationship between coach–athlete attachment and thriving. In line with the aims of Study 1, the hypotheses are firstly, a secure coach–athlete attachment relationship will have a positive association with thriving, while an insecure avoidant and anxious coach–athlete attachment relationship will have a negative association with thriving. Secondly, we hypothesize that basic psychological needs satisfaction will mediate the associations between secure coach–athlete attachment and insecure (anxiety and avoidance) coach–athlete attachment and thriving.

Study 2 aims to provide a preliminary examination of the predictive effects of parental attachment (mother and father) on thriving and competition performance within the sport of gymnastics. Gymnasts are often placed in competitive environments that require them to cope with various psychological demands and pressures (e.g., expectations) at an early age (Mellalieu et al., 2009; Jacobs et al., 2017). As such, the anxiety and fear associated with gymnasts’ competition may activate the need for parental security in order to buffer the negative effects associated with not being able to perform well in the sport (Feeney and Collins, 2015a). Additionally, by conducting the study in a specific sport and situating the experience of thriving within a competition, we could record objective performance via judges’ scores. In so doing we were able to address a limitation of previous thriving literature pertaining to the need to consider the role of match/competition outcome with thriving (see, Brown et al., 2021a). Therefore, based on previous research, we first hypothesize that gymnasts’ secure attachment with their mother and/or father will positively predict the experience of thriving at the competition and an insecure attachment with mother and/or father will negatively predict the experience of thriving at the competition. Secondly, we hypothesize that a gymnast’s secure attachment with his/her mother and/or father will positively predict competition performance and an insecure attachment will negatively predict competition performance. Thirdly, we hypothesize that a gymnast’s experience of thriving at the competition will be positively associated with competition performance.

## Study 1

### Method

#### Participants

The sample included 290 Swedish athletes (138 female and 152 male) ranging in age from 11 to 46 years old and with a mean age of 18.46 (*SD_Age_* = 4.54). Participants were involved in a variety of individual and team sports (e.g., football, basketball, floorball, ice hockey, badminton, golf, and gymnastics) and represented their sports at various levels of performance including recreational (1.0%), club (2.1%), regional (64.1%), national (29.3%) and international (3.1%) levels (0.3% did not specify level). Furthermore, participants trained on average 9.2 h per week (*SD* = 6.00) and reported an average coach–athlete relationship length of 2.8 years (*SD* = 2.39).

#### Procedures

Ethical approval to conduct this study was granted by the Regionala Etikprövningsnämnden i Umeå. Upon ethical approval, sport organizations and sports clubs were contacted via phone and/or email using both purposeful and convenience sampling techniques with information regarding the study and to elicit their athletes’ participation. A cross sectional, questionnaire-based design was employed. Upon consent, one of two methods for data collection was adopted. First, a date and time for the research team to visit the sports clubs closest to the first author were arranged. Upon meeting the participants at the beginning of a training session, the aims and objectives of the study were explained and written consent was obtained. The confidentiality and anonymity of the study were outlined, and participants were informed of their right to withdraw from the study by contacting the author and providing their unique code. A multi-section questionnaire was then distributed in paper and pencil format, and participants were reassured of the anonymity and confidentiality of their responses. Participants were asked to complete the questionnaire independently from their coach and peers, and members of the research team were on hand to supervise and respond to any queries. This process took approximately 20 min. For those athletes’ who could not be contacted face to face, a second method of data collection that involved a web-based survey was utilized. Sport clubs and organizations were asked to distribute the web-based survey link they were sent by the research team to their athletes. The web-based survey explained the purpose, participants’ ethical rights, as well as instructions on how to complete the questionnaire online. Upon consent, the multi-section questionnaire became available. Following completion, the participants’ data were electronically sent to a secure database for analysis.

#### Measures

The following measures were used in the present study. All items were translated to the Swedish language using a parallel back translation process.

##### Coach–Athlete Attachment

The Coach–Athlete Attachment Scale (Davis and Jowett, 2013) contains 19 items designed to measure an athlete’s secure and insecure attachment styles toward their principle sports coach. Specifically, five items measured athletes’ secure attachment (e.g., “I know I can rely on my coach”), seven items measured athletes’ insecure anxious attachment (e.g., “I worry that I won’t fulfil my coaches’ expectations”), and seven items measured athletes’ insecure avoidant attachment (e.g., “I do not turn to my coach for reassurance”). Participants were asked to indicate the extent to which they agreed with each statement on a seven-point Likert scale (1 = *strongly disagree*, 7 = *strongly agree*) in relation to how they felt toward their principle sports coach within the last month. Evidence for the validity and reliability of this instrument has been provided by Davis et al. (2013) and Davis and Jowett (2014).

##### Basic Psychological Need Satisfaction

The 20-item Basic Need Satisfaction in Sport Scale (BNSSS; Ng et al., 2011) was utilized to measure athletes’ basic psychological needs satisfaction. Specifically, 10 items measured athletes’ autonomy satisfaction (e.g., “In my sport, I get opportunities to make choices”), five items measured competence satisfaction (e.g., “I am skilled at my sport”), and five items measured relatedness satisfaction (e.g., “In my sport, I feel close to other people”). Participants were asked to respond on a seven-point Likert scale (1 = *Not true at all*, 7 = *very true*) in relation to how they felt within the last month. Ng et al. (2011) provided support for the factor structure of the scale and its internal consistency. As in previous research (e.g., Jowett and Shanmugam, 2016), a composite approach (i.e., a global factor) was implemented for basic psychological need satisfaction, with average subscale scores for autonomy satisfaction, competence satisfaction, and relatedness satisfaction used as observed values for a latent need satisfaction variable. The Cronbach alpha values for the autonomy satisfaction, competence satisfaction, and relatedness satisfaction subscales were 0.87, 0.88, and 0.92, respectively.

##### Thriving

Participants were asked to provide evaluations of their subjective performance and well-being to assist in identifying sport performers who thrived (cf. Brown et al., 2017a). Taking subjective performance first, this was measured by asking participants to rate their satisfaction with personal sporting performance over the past month on an 11-point Likert scale ranging from 0 = *totally dissatisfied* to 10 = *totally satisfied* (Levy et al., 2011; Arnold et al., 2017; Brown et al., 2018). In line with Brown et al.’s (2018) conceptualization of thriving in sport as well as Ryan et al.’s (2013) recognition of differentiated approaches to understanding well-being, separate measures were used to assess hedonic and eudaimonic well-being. The indicator of hedonic well-being in this study was the positive affect scale from the Positive and Negative Affect Schedule Short Form (I-PANAS-SF; Thompson, 2007). Specifically, participants were asked to report the extent to which they experienced five emotional descriptors (viz., active, alert, attentive, determined, inspired) during their sporting encounters over the past month on a five-point Likert scale ranging from 1 = *never* to 5 = *always*. To indicate eudaimonic well-being, the Subjective Vitality Scale (SVS; Ryan and Frederick, 1997) was used, with participants reporting the extent to which they experienced aliveness and energy in their sporting encounters over the past month. Specifically, participants were asked to respond to four items from the SVS (e.g., “I felt alive and vital”) on a six-point scale ranging from 1 = *not at all true* to 6 = *very true*. Subscale scores for positive affect and subjective vitality were used as observed values (alongside subjective performance) for a latent thriving variable. The Cronbach alpha values were 0.85 for the positive affect subscale and 0.93 for the subjective vitality subscale.

##### Data Analysis Plan

Analyses were conducted using SPSS 25 (IBM, 2017) and MPlus 8.4 (Muthén and Muthén, 2019). SPSS 25 was used to screen for the proportion of missing data, univariate and multivariate outliers, and to compute the subscale scores for autonomy satisfaction, competence satisfaction, relatedness satisfaction, subjective vitality, and positive affect. In addition, scores were computed for the components of attachment to report the level of attachment athletes felt toward their coaches. Mplus 8.4 was used to determine the fit of the measurement model, calculate descriptive statistics for and correlations between latent constructs, and to examine the mediation model using a structural equation modeing framework. All analyses in Mplus 8.4 were conducted using a maximum likelihood estimation with robust standard errors (MLR) to account for any non-normality within the data and any missing values (Muthén and Muthén, 2015); Mplus syntax for the analyses can be viewed in the Electronic Supplementary Resources.

The raw data set was initially screened for univariate outliers by comparing reported values to the minimum and maximum permissible scores for each of the scale items, with any inadmissible values replaced with a missing data value. Next, the proportion of missing data within the data set was assessed and cases with large amounts of missing data (>10%) were removed (cf. Hair et al., 2010). In instances where a case was missing data on a small number of items and data were deemed to be missing at random, the expectation–maximization algorithm was used to impute the missing values (cf. Tabachnick and Fidell, 2013). The item-level data were then averaged to create the respective subscale scores, with the subscale scores then used to identify any multivariate outliers; outliers were determined using the Mahalanobis distances with *p* < 0.001 (Tabachnick and Fidell, 2013). Following the completion of data screening, the subscale scores were considered as observable indictors of the latent factors for need satisfaction and thriving.

The measurement model was constructed with each of the latent variables allowed to freely correlate. The adequacy of the measurement model was determined via interpretation of model fit indices and parameter estimates (see Gunnell et al., 2016). Model fit indicies included the Comparative Fit Index (CFI) and Tucker-Lewis Index (TLI) with values *close to or above* 0.90 interpreted as acceptable, and Standardized Root Mean Square Residual (SRMR) and Root Mean Square Error of Approximation (RMSEA) with values *close to or below* 0.08 considered as acceptable (see, Marsh et al., 2016). Parameter estimates were examined to determine whether items were behaving as had been intended with acceptable standardized factor loadings of above 0.30 and statistically significant (*p* < 0.05 and confidence intervals did not cross zero; Brown, 2006). On the occurrence of inadequate global model fit, modification indices were used to identify areas of possible ill fit (e.g., where a specific restriction on the model is related to global misfit) and then the researchers discussed any proposed modifications in the context of previous research and theoretical knowledge. The measurement model was also used to compute the mean and standard deviation values for each of the latent constructs and the correlations between them.

**Figure 1:**
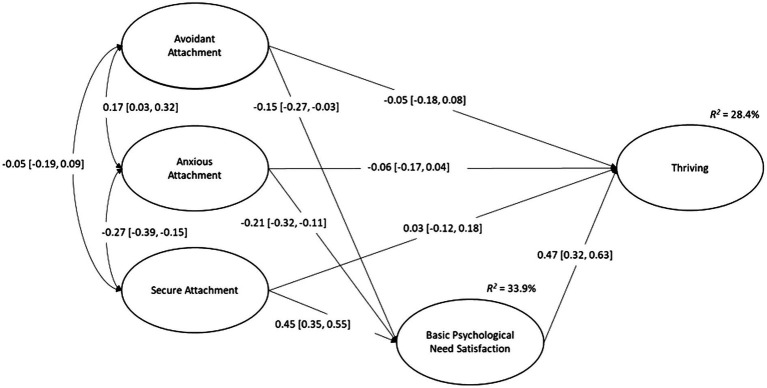
Latent path model displaying the mediation model with attachment styles, basic psychological need satisfaction, and thriving. Standardized parameter estimates are displayed with the 95% confidence interval in parentheses.

To examine the potential mediating effect of need satisfaction on the relationships between the attachment styles and thriving, two latent path models were constructed. The first included the data for attachment styles and thriving, with thriving regressed on the styles to establish whether any direct, predictive paths existed (Model 1). Need satisfaction was then added in the second model, along with indirect paths for the predictive effect of attachment style on thriving via need satisfaction (see Figure 1; Model 2). The direct and indirect effects were interpreted using the unstandardized and standardized factor loadings, and statistical significance (*p* < 0.05 and confidence intervals did not cross zero). The statistical significance of the indirect effects was also interpreted using bias-corrected 95% confidence intervals^1^ (MacKinnon et al., 2004).

## Results

### Data Screening

Following data screening, four cases were removed from the data set for missing greater than 10% of data, and 17 multivariate outliers were excluded; no univariate outliers were identified. Therefore, the final sample size for the measurement model and mediation analysis was 269.

### Measurement Model

The measurement model demonstrated acceptable fit based on CFI, TLI, RMSEA, and SRMR values ($\mathrm{MLR}\chi_{\left(265\right)}^{2}$= 593.105, *p <* 000; CFI = 0.916; TLI = 0.905; RMSEA [90% CI] = 0.068 [0.061,0.075]; SRMR = 0.074). All standardized loadings were above the recommended threshold of 0.300 and statistically significant. The descriptive statistics for, and correlations between, each of the latent variables are presented in Table 1.

**Table 1:** Descriptive statistics and correlations for avoidant attachment, anxious attachment, secure attachment, need satisfaction, and thriving.

S. No.	Variable	1	2	3	4	*M*	*SD*
		*r* (95%CI)	*r* (95%CI)	*r* (95%CI)	*r* (95%CI)		
1.	Avoidant attachment	–				3.55	1.38
2.	Anxious attachment	0.171^*^ [0.026, 0.317]	–			2.45	1.03
3.	Secure attachment	−0.050 [−0.194, 0.093]	−0.271^***^ [−0.389, −0.154]	–		4.96	1.32
4.	Need satisfaction	−0.208^**^ [−0.341, −0.075]	−0.362^***^ [−0.459, −0.265]	0.513^***^ [0.420, 0.607]	–	4.32	0.72
5.	Thriving	−0.165^*^ [−0.317, −0.013]	−0.251^***^ [−0.375, −0.127]	0.296^***^ [0.173, 0.419]	0.525^***^ [0.406, 0.645]	6.61	1.01

* *p* < 0.05;

** *p* < 0.01;

*** *p* < 0.001.

### Mediation Analysis

The results from Model 1 indicate that significant predictive relationships existed between anxious attachment and thriving (βˆ_ANX_= −0.152, *z =* −2.126, *p* =0.033, ${\hat{\beta }}_{\mathrm{ANX}}^{\mathrm{standardized}}$ = −0.155), and between secure attachment and thriving (βˆ_SECUR_ = 0.192, *z =* 3.616, *p* <0.001, ${\hat{\beta }}_{\mathrm{SECUR}}^{\mathrm{standardized}}$ = 0.252); however, a non-significant prediction was found for avoidant attachment and thriving (βˆ_AVOID_ = −0.080, *z =* −1.366, *p* =0.172, ${\hat{\beta }}_{\mathrm{AVOID}}^{\mathrm{standardized}}$ = −0.110). When need satisfaction was added as a mediator in Model 2, the relationships between the five constructs were in the expected direction. However, the direct paths from the attachment styles to thriving were non-significant: avoidant attachment and thriving (βˆ_AVOID_ = −0.039, *z =* −0.794, *p* = 0.427, ${\hat{\beta }}_{\mathrm{AVOID}}^{\mathrm{standardized}}$ = −0.054), anxious attachment and thriving (βˆ_ANX_ = −0.059, *z =* −1.091, *p* = 0.275, ${\hat{\beta }}_{\mathrm{ANX}}^{\mathrm{standardized}}$ = −0.061), and secure attachment and thriving (βˆ_SECUR_ = 0.025, *z =* 0.447, *p* = 0.655, ${\hat{\beta }}_{\mathrm{SECUR}}^{\mathrm{standardized}}$ = 0.033). Need satisfaction was a significant, positive predictor of thriving (βˆ_NS_ = 0.665, *z =* 4.047, *p* <0.001, ${\hat{\beta }}_{\mathrm{NS}}^{\mathrm{standardized}}$ = 0.475). The relationships between attachment styles and need satisfaction were significant and in the predicted direction: avoidant attachment and need satisfaction (βˆ_AVOID_ = −0.078, *z =* −2.410, *p* = 0.016, ${\hat{\beta }}_{\mathrm{AVOID}}^{\mathrm{standardized}}$ = −0.149), anxious attachment and need satisfaction (βˆ_ANX_ = −0.150, *z =* −3.994, *p* < 0.011, ${\hat{\beta }}_{\mathrm{ANX}}^{\mathrm{standardized}}$ = −0.215), and secure attachment and need satisfaction (βˆ_SECUR_ = 0.245, *z =* 6.710, *p* < 0.001, ${\hat{\beta }}_{\mathrm{SECUR}}^{\mathrm{standardized}}$ = 0.447). Significant, indirect effects were found for each of the attachment styles on thriving, with avoidant attachment (−0.052, *p* = 0.033, B-C 95% CI [−0.120, −0.013]) and anxious attachment (−0.100, *p* = 0.005, B-C 95% CI [−0.193, −0.044]) shown to have negative effects, and secure attachment to have a positive effect (0.163, *p* < 0.001, B-C 95% CI [0.094, 0.268]). As such, the results suggest that need satisfaction fully mediates the effects of attachment styles on thriving. However, the variance explained in need satisfaction (*R*^2^ = 33.9%) and thriving (*R*^2^ = 28.4%) suggests that unmeasured variables are likely to exist which also contribute to the prediction of these constructs. The final model is shown in Figure 1.

## Study 2

### Methods

#### Participants

A sample of 40 (female *n =* 34; male *n =* 6) Swedish gymnasts aged between 11 and 25 (*M*age = 14.30, *SD =* 2.62) volunteered to take part in the study. The participants described competing across junior (5%), senior (12.5%), regional (67.5%), or 'other' (15%) levels, and trained on average for 11.28 h per week (*SD* = 4.37).

#### Procedure

A prospective design was employed for Study 2 using a purposeful sampling technique. Following approval from the Regionala Etikprövningsnämnden i Umeå, the Swedish Gymnastics Federation were contacted by email and telephone outlining the aims and objectives of the study and were asked to participate by providing contacts for and access to clubs across Sweden that they thought suitable for this project. Suggested gymnastic clubs were then contacted by email and/or telephone and a date and time for the first author to visit and discuss the project with coaches, athletes, and parents were arranged. Upon contact, the purpose and voluntary nature of the study were explained. Informed consent was obtained from participants willing to participate, and parental consent was obtained from those who were under the age of 18. Upon receiving informed and parental consent, an additional visit during a standard training session was arranged at least two weeks prior to an upcoming national competition, where participants were asked to complete a questionnaire containing demographic information and questions relating to their attachment relationship with their mother and father. Participants were asked to complete the questions independently from their parents and peers. To reduce potential problems associated with understanding and readability in the sample, participants were encouraged to ask questions to the research team present if they were unsure of the meaning of any items. At the time of their respective competitions, participants were required to complete measures of well-being 45 min before their performance and provide an indication of subjective performance within 30 min of competing. Each competition routine was video-recorded by a member of the research team.

#### Measures

##### Parental Attachment

Athletes’ attachment relationship with their parents, including both mother and father, was measured with the Swedish version of the Inventory of Parent and Peer Attachment (IPPA; Armsden and Greenberg, 1987). The IPPA contains 25 items across three subscales that evaluates the degree of mutual trust (10 items; e.g., “my mother/father respects my feelings”), quality of communication (nine items, e.g., “I tell my mother/father about my problems and troubles”) and prevalence of anger and alienation from mothers and fathers (six items; e.g., “I feel angry with my mother/father”). These questions are repeated for each attachment relationship (e.g., mother, father). Participants are asked to rate each item using a five-point Likert scale (1 = *almost never or never* to 5 = *almost always or always*) to indicate the degree to which the items are true. Secure attachment is indicated by a combination of trust and communication; therefore, a secure attachment score was derived from averaging trust and communication ratings. Insecure attachment is indicated by high ratings of alienation. Sound psychometric properties have been demonstrated within the initial validation of the IPPA scale and have since been used in an extensive number of studies including with sport samples (Li et al., 2016). Cronbach’s alpha scores for mother secure and insecure attachment were 0.59 and 0.62 and for father secure and insecure attachment 0.65 and 0.50, respectively.

##### Thriving

Participants were asked to provide evaluations of their subjective performance and well-being to assist in identifying sport performers who thrived in the present study (Brown et al., 2017a). The scales for both subjective performance and well-being have been described within the measures section of Study 1; however, the subjective performance measure was amended in this study to ask participants how they felt they performed during their routine, rather than over the past month. As such, the pre-routine well-being assessment provided a general indication of how participants were feeling when arriving at the competition (i.e., overall well-being over the past month) and the post-routine performance assessment offered a specific evaluation of the performance delivered during that competition. These ratings have been used together to provide a general indication of levels of thriving at the competition.

##### Competition Performance

Participants’ competitive routines were video-recorded by the first author during a national competition selected by the participants’ gymnastics club. In light of the fact that not every gymnast had competed at the same event, with the same set of judges, the gymnasts’ routines were marked by a consistent panel of professional judges certified with the Swedish Gymnastics Federation and the International Gymnastics Federation (FIG). Specifically, in line with FIG’s code of point’s guidelines and scoring system, two male judges were selected to mark the male gymnasts’ routines and two female judges were selected to mark the female gymnasts’ routines. Marks were awarded for both execution on a scale between 0 (*did not perform*) to 10 (*perfect and faultless*) and for difficulty on a scale between 0 (*not difficult*) to 6 (*high difficulty*). Mean judge scores were calculated for each participant, which represented each participant’s overall performance score. All judges were blind to the nature of the study and provided their scores independently of the other judges.

##### Data Analysis

Owing to the relatively small sample size, separate analyses were conducted to examine the effects of mother and father attachment. As with Study 1, SPSS 25 and Mplus 8.4 were used to conduct the data analysis, with the MLR estimator used to account for any non-normality and missing values within the data. Data were screened for cases with a high proportion of missing data (> 10%), univariate and multivariate outliers using the same criteria as Study 1. Prior to checking for multivariate outliers, averaged values were computed for mother/father trust, mother/father communication, mother/father alienation (i.e., insecure attachment), subjective vitality, and positive affect; values for trust and communication were then averaged to create a composite score for mother/father secure attachment. To derive a singular score for thriving, FScores were computed in Mplus from a measurement model including subjective performance, subjective vitality, and positive effect as indicators of a latent, thriving variable (see, Brown et al., 2020a). Manifest path models were then specified with competition performance and thriving regressed on mother/father secure attachment and mother/father insecure attachment. Regression paths were interpreted using the unstandardized and standardized factor loadings, and statistical significance (*p* < 0.05 and confidence intervals did not cross zero).

## Results

### Data Screening

Six cases were removed from the mother attachment analysis due to high levels of missing data; no univariate or multivariate outliers were identified. The final sample size for this analysis was 34. Seven cases were removed from the father attachment analysis due to high levels of missing data; no univariate or multivariate outliers were identified. The final sample size for this analysis was 33.

### Manifest Path Analysis

**Figure 2:**
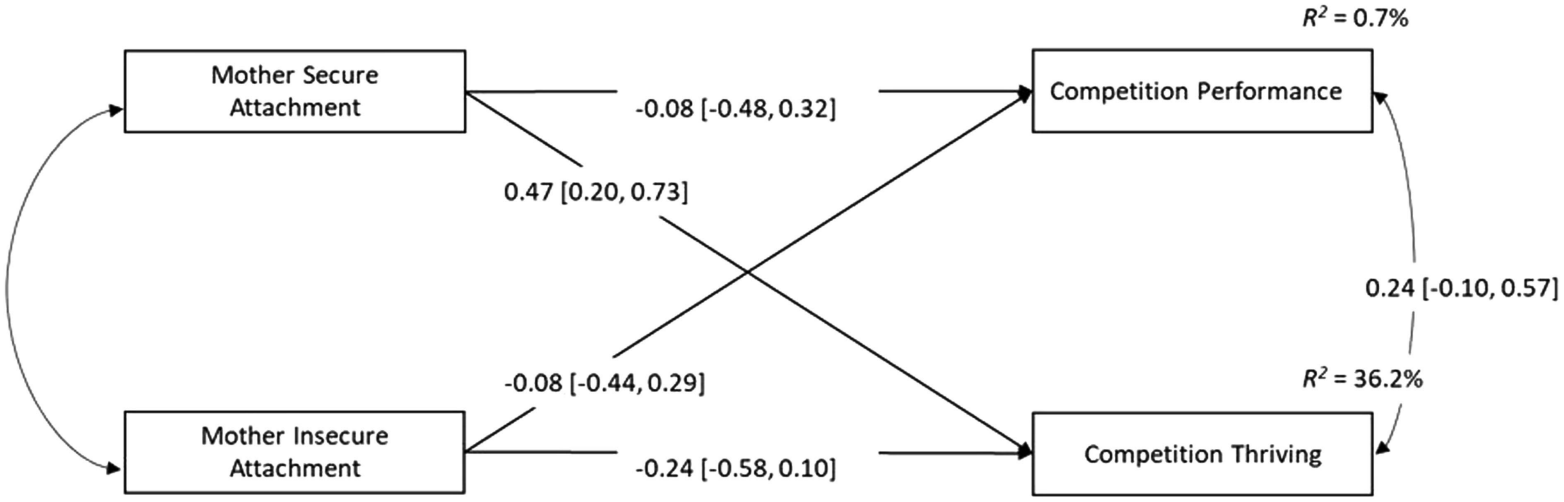
Manifest path model displaying the relationships between mother secure attachment, mother insecure attachment, competition performance, and competition thriving. Standardized parameter estimates are displayed with the 95% confidence interval in parentheses.

**Figure 3:**
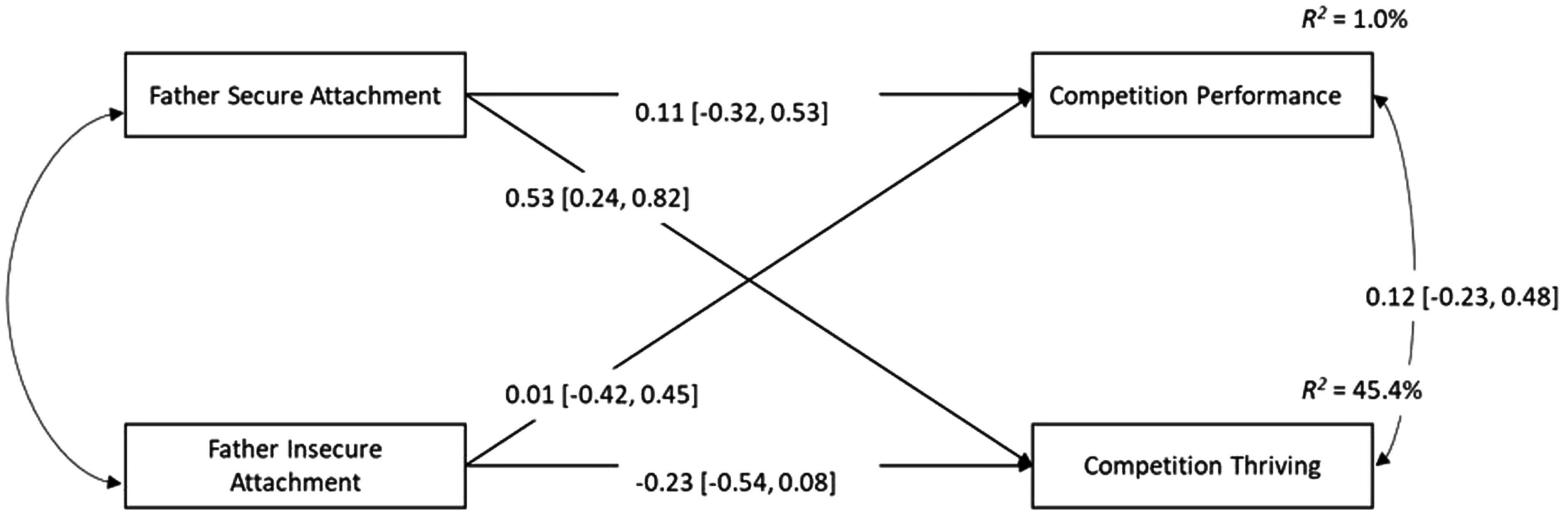
Manifest path model displaying the relationships between father secure attachment, father insecure attachment, competition performance, and competition thriving. Standardized parameter estimates are displayed with the 95% confidence interval in parentheses.

Descriptive statistics and correlations between variables for the mother attachment and father attachment analyses are displayed in Table 2. These results suggest that competition performance was not related to any of the other variables in either the mother or father attachment data sets. Path models were drawn to examine the predictive effects of mother/father secure and insecure attachments on thriving and objective performance (see Figures 2, 3). The results suggest that thriving was predicted by mother secure attachment (βˆ_MSECUR_ = 1.501, *z =* 3.182, *p* = 0.001, ${\hat{\beta }}_{\mathrm{MSECUR}}^{\mathrm{standardized}}$ = 0.466), while controlling for the effect of mother insecure attachment. Mother insecure attachment did not predict thriving, and neither secure nor insecure attachment predicted competition performance. The path model for father attachment suggested that, when controlling for the effects of insecure attachment, secure attachment was a positive predictor of thriving (βˆ_FSECUR_ = 1.415, *z =* 3.316, *p* =0.001, ${\hat{\beta }}_{\mathrm{FESCUR}}^{\mathrm{standardized}}$ = 0.532). No other predictive paths were statistically significant. Readers are encouraged to interpret these results cautiously, given the large confidence intervals and associated standard errors.

**Table 2:** Descriptive statistics and correlations for secure attachment, insecure attachment, competition performance, and thriving.

S. No.	Variable	1	2	3	4	*M*	*SD*
		*r* (95%CI)	*r* (95%CI)	*r* (95%CI)	*r* (95%CI)		
	*Mother attachment*						
1.	Secure attachment	–				3.94	0.26
2.	Insecure attachment	−0.388^**^ [−0.638, −0.138]	–			1.70	0.57
3.	Competition performance	−0.049 [−0.407, 0.310]	−0.047 [−0.377, 0.284]	–		7.69	2.84
4.	Thriving^a^	0.559^***^ [0.364, 0.754]	−0.422^**^ [−0.714, −0.129]	0.176 [−0.173, 0.525]	–	0.00	0.83
	*Father attachment*						
1.	Secure attachment	–				3.90	0.30
2.	Insecure attachment	−0.483^***^ [−0.721, −0.246]	–			1.63	0.49
3.	Competition performance	0.100 [−0.260, 0.461]	−0.038 [−0.407, 0.330]	–		7.65	2.88
4.	Thriving^a^	0.643^***^ [0.434, 0.853]	−0.487^**^ [−0.793, −0.181]	0.153 [−0.203, 0.510]	–	0.00	0.81

a Subscales for thriving were standardized when computing the FScores, resulting in the mean value of 0.00.

** *p* < 0.01;

*** *p* < 0.001.

## Discussion

The overarching aim of this paper was to contribute to the emerging research area of thriving in sport by examining “if” and “how” relationships with significant others, such as parents and/or sports coaches, enable (or hinder) athlete thriving. As such, this paper presents the findings from two studies. Study 1 aimed to: (1) examine the relationship between coach–athlete attachment and thriving across a variety of sports; and (2) examine the mediating effects of basic psychological need satisfaction on the relationship between coach–athlete attachment and thriving. Study 2 examined the predictive effects of parental attachment (mother and father) on thriving and in-competition performance within the sport of gymnastics.

Specifically, in Study 1 it was hypothesized (H1) that a secure coach–athlete attachment relationship would have a positive association with thriving, while an insecure (anxious and avoidance) coach–athlete attachment relationship would have a negative association with thriving. In line with these hypotheses, positive associations were found between athletes’ secure attachment and thriving and a negative association between athletes’ anxious attachment and thriving. Contrary to our expectations, no significant associations were found for athletes’ avoidant attachment and thriving. This suggests that athletes who perceive their coach–athlete relationship to be characterized by emotional closeness, trust, and support and possess positive IWMs of their coach (i.e., optimistic expectations, thoughts, and feelings) as well as themselves (i.e., positive self-image), were found to thrive. On the other hand, those athletes who perceived their relationship with their coach to be characterized by uncertainty and a fear of rejection do not thrive. Working models of attachment are central to social perception processes (Collins et al., 2006), which may explain why athletes with varying attachment styles experience differential outcomes associated with thriving, which is measured subjectively.

Working models of attachment are highly accessible cognitive–affective structures that shape how individuals construe their social experiences (Collins and Allard, 2001). For example, secure individuals have positive self-images and optimistic expectations of others, this allows them to remain positive about themselves and interpret their relational experiences and associated outcomes in relatively favorable ways (Collins et al., 2006). In consideration of the findings of the present study, the positive IWMs may provide the mechanism underlying athletes’ positive subjective experiences of performance and well-being when participating in their sport. In contrast, insecure working models represent a cognitive vulnerability that predisposes individuals to perceive their relationship and associated outcomes less favorably (Collins et al., 2006). In the present study, athletes with an insecure anxious attachment to their coach may have also possessed negative IWMs that inhibit positive subjective experiences of performance, as well as well-being. As for the nonsignificant findings with avoidant attachment, this is in contrast to previous research in sport whereby an avoidant attachment style toward a sports coach was found to be linked with greater dysfunctionality and lower levels of well-being (Davis and Jowett, 2010, 2014).

Taken together, these findings point to the importance of identifying specific needs and goals of individuals with different attachment styles and exploring their role in shaping intra- and interpersonal experiences. As such, the second hypothesis of Study 1 (H2) proposed that basic psychological need satisfaction would mediate the association between coach–athlete attachment (i.e., secure, anxious, and avoidant) and thriving. In support of the hypothesis, findings from Study 1 provide initial evidence that avoidant and anxious coach–athlete attachment are associated with limited thriving via a perceived lack of need satisfaction. That is, athletes with an avoidant or anxious attachment style who perceive their needs (i.e., autonomy, competence, and relatedness) are not being satisfied are likely to experience a less thriving in their sport. On the contrary, the findings outline that a secure coach–athlete attachment is associated with thriving via greater perceived need satisfaction.

Overall, these findings appear to suggest that athletes can thrive when their coach is engaging in coaching behaviors that create an environment in which the athlete feels their needs are being satisfied (Mageau and Vallerand, 2003). This is of particular importance, especially for those athletes with an anxious or avoidant attachment style, as basic needs satisfaction may alleviate some levels of dysfunctionality and promote thriving. Further, previous research highlights that basic need satisfaction can mediate the relationship between an athletes’ avoidant attachment to their coach and well-being (Felton and Jowett, 2013). The findings also lend support to the contention that basic psychological needs satisfaction is an underpinning process variable through which social-contextual factors (i.e., coaches) can impact thriving (Brown et al., 2017a).

The social factors examined in Study 2 centered on the role of parents, whereby it was first hypothesized that gymnasts’ secure attachment toward their mother and/or father would positively predict the experience of thriving at a competition, while an insecure attachment toward a mother and/or father would negatively predict thriving. The findings partially supported our hypothesis, as thriving was predicted by mother and father secure attachment only; mother and father insecure attachment did not significantly predict thriving. Therefore, perceived security in the mother–child and father–child relationship emerges as being particularly important for athletes’ optimal functioning and is reflected in athletes’ subjective well-being (i.e., positive affect and subjective vitality) and performance. Moreover, these findings sit well alongside research highlighting that a secure attachment relationship to parents is associated with subjective and psychological well-being (e.g., happiness and growth; Felton and Jowett, 2013, 2017). It also extends research that has identified the significant role that parental attachment plays in sport by focusing on identifying athletes’ attachment relationship to their mother and father independently of their global attachment representations. It is noteworthy, however, that the association between an athlete’s insecure attachment to their mother and father and thriving was nonsignificant. A potential explanation of the finding may relate to the observations noted in Study 1 where other potential variables (e.g., basic psychological needs satisfaction) serve as mechanisms by which an athletes’ insecure attachment to their mother or father is linked to thriving. That said, this conjecture warrants further investigation.

Finally, it was hypothesized that a gymnast’s secure attachment with their mother and/or father would positively predict competition performance, while an insecure attachment would negatively predict competition performance. Our findings suggest that competition performance was not related to either mother or father attachment. One possible explanation for this could be that gymnasts’ attachment to their parents was measured on a global level, rather than on a contextual level. Research indicates that individuals are capable of developing context specific attachment bonds with parents, especially when the context elicits parental belief systems in regard to their child’s ability, success, and failures (Ames, 1992; Lai and Carr, 2018). In particular, within achievement contexts such as sport, parents may demonstrate maladaptive parenting practices. Specifically, parents have been observed offering either more or less affection, accessibility, and recognition, depending upon how the child performs and meets their expectations. This is known as parental conditional regard (PCR; Assor et al., 2014). Parents’ subjective evaluation of their children’s successes and failures has the potential to serve as influential “contextual cues” that shape children’s IWMs, and therefore their attachment beliefs within a given context (Lai and Carr, 2018). As such, it is possible that within the present study gymnasts held contextual attachment representations toward their parents that were not evident through the measurement of attachment on a global level. This potential explanation warrants further investigation in future research using more refined measurement techniques.

Taken collectively, the findings from both studies provide initial evidence that secure close attachment relationships in sport are fundamental to athletic thriving. Moreover, our findings align with Feeney and Collin’s (2015a) conceptual suggestion that humans can thrive through secure (close, caring) relationships both during adversity (e.g., stress of competition) and in the absence of adversity (e.g., during training). Moreover, this is the first study that has attempted to explore athletes’ attachment relationships as contextual enablers of thriving within the context of sport. Similarly, the present study is the first to extend the attachment research literature by examining the role of parental attachment in relation to athletes’ objective performance in a competitive environment. Examining multiple relationships enables the development of a more comprehensive picture outlining how relationships with significant others both in general and within an intense competitive environment influence athletes’ thriving.

Notwithstanding the studies’ strengths, limitations are inevitable and should guide future research. The first limitation stems from the cross-sectional nature of Study 1, which introduces common method variance/bias and prevents inferences of causality. Although the research extends beyond a cross-sectional design in the prospective research design of Study 2, the nature of the observational data (i.e., limited control) precludes the investigation of cause and effect relationships. Further research is warranted to examine the model proposed within Study 1 from a longitudinal perspective, to determine the temporal precedence and causal nature of the proposed relationships. Although Study 1 provides initial information for the development of interventions aiming to enhance athletic thriving through the satisfaction of basic psychological needs, it remains unclear as to whether a specific need may be more important than another. Future research should consider examining the sub-domains of basic needs satisfaction separately as well as potential interactions of combined individual needs. Furthermore, in the present study, athletes’ basic psychological needs were assessed in respect to sport in general. Future research could also consider assessing satisfaction of basic psychological needs with respect to the coach. In the present study, this would have complimented other measures (e.g., attachment relevant to the coach). Finally, in regard to Study 1, the sample was comprised of both individual and team sports, as well as a wide range of ages and levels of participation. This potentially creates issues with biased estimates and generalizability of the findings. To address potential limitations regarding heterogeneity of the sample, the subsequent study chose to focus on a sample of greater homogeneity.

Second, Study 2 examined the relationship between parent–child attachment and thriving within the context of gymnastics given the heightened experiences of stress experienced by these athletes. In doing so, we recognize that the findings may not be applicable to all youth sport contexts and encourage readers to reflect on the relevance of these findings to their sporting environments. Third, the reliability scores for secure and insecure attachment to mother and father did not quite meet the criteria (>0.7), although this may be relative to the sample size and the research design. Fourth, purpose of Study 2 was to provide preliminary data within a specific sport and situating the experience of thriving within a competition, where we could also record objective performance via judges’ scores. In doing so we have made steps in addressing a limitation of previous thriving literature pertaining to the need to consider the role of match/competition outcome with thriving (see, Brown et al., 2021a). That said, to improve power in future work and to reduce the risk of false positive and false negative findings, we encourage researchers to consider additional sports beyond gymnastics, where access to larger groups of participants within a particular performance category and/or age groups is feasible.

Lastly, the relationship between coach–athlete attachment and thriving, as well as parent–child attachment and thriving, was examined separately; therefore, it was not possible to draw inferences regarding the hierarchy of these attachment relationships. To elaborate, while adolescents and adults maintain attachment bonds with multiple figures (e.g., parents, coaches, peers), they also have a consistent order of preference for whom they would seek out during times of need and/or stress (Bowlby, 1969/1982). Future research would benefit from measuring coach–athlete and parent–athlete relationships simultaneously while identifying an order of preference, particularly during an intense and potentially stressful environment, such as competition where the attachment system is likely to be activated (Ainsworth et al., 1978). Furthermore, by studying multiple relationships simultaneously, we can also identify if athletes’ attachment styles toward their coach are relatively independent of the attachment style an athlete reports toward their parent(s). This is an important question, given that the adolescent and attachment research literature outlines critical arguments surrounding the stability of attachment across domains (Weiss, 1982; Zimmermann, 2004).

The findings presented in this study offer a number of important practical implications. First, the current study may guide the development of interventions that facilitate thriving by targeting coaches with the aim of systematically and deliberately implementing coaching strategies that address and satisfy athletes’ basic psychological needs. This is especially important to help support athletes with an insecure anxious or avoidant attachment style. As such, it is possible that sport psychologists and organizations at a local level could work with coaches to create environments which are underpinned with greater autonomy supportive behaviors versus controlling behaviors. Coaches displaying controlling behaviors are likely to induce athletes’ experience of feeling fearful, upset, nervous, and hostile; controlling behaviors have the potential to interrupt a secure attachment bond that is required for thriving to occur (Bartholomew et al., 2011; Felton and Jowett, 2015). Secondly, if coaches are able to satisfy their athletes’ basic psychological needs through implementation of more autonomy supportive behaviors, it is possible that this could provide a buffer against neglectful parent–athlete relationships (insecure attachments) and support the athlete to thrive during adversity in the context of competition (Feeney and Collins, 2015a). The findings from the current studies highlight the potentially important role of the parent and coach, in athlete thriving. Future interventions could aid the development of sport specific education programs that guide parent and coach behavior that also acknowledges the importance of positive relations (secure attachments), in which parents and coaches consistently communicate trust, reassurance, support, and acceptance (Feeney and Collins, 2015b). While an athlete with an insecure attachment may be difficult to coach due to their lack of connection (avoidant) or too much needed connection (anxious), attempting to deliberately enhance the athletes trust, respect, and commitment overtime may facilitate changes in their internal working models (IWMs) that allow the athlete to develop a positive relationship. Afterall, the aim of sport is also to provide equal opportunities, whereby all athletes’ get the same quality of training (Jowett and Felton, 2014).

## Conclusion

The two studies presented shed light on a relatively unexplored area of thriving in athletes by providing significant evidence on the role of attachment relationships to significant others (e.g., parents and/or sports coaches) in influencing thriving. Further, the role of basic psychological needs satisfaction in facilitating thriving, especially for those with an insecure anxious or avoidant attachment style, forwards an important consideration for coaches, parents, and practitioners. These findings can inform the development of interventions that optimize the contextual enablers of thriving within sport.

## Data Availability Statement

The raw data supporting the conclusions of this article will be made available by the authors, without undue reservation.

## Ethics Statement

The studies involving human participants were reviewed and approved by Regionala Etikprövningsnämnden i Umeå. Written informed consent to participate in this study was provided by the participants’ legal guardian/next of kin.

## Author Contributions

LD designed Study 1 and Study 2, worked with HG on data recruitment, collected data and prepared the manuscript for publication. DB analysed the data for both Study 1 and Study 2 and together with LD prepared the manuscript for publication. RA worked together with LD and HG in designing Study 1 and Study 2 and contributed to the writing of the manuscript. HG worked together with LD and RA in designing Study 1 and Study 2. HG was also responsible for the Ethics application, translation of questionnaires and informed consent as well as data recruitment. All authors contributed to the article and approved the submitted version.

## Conflict of Interest

The authors declare that the research was conducted in the absence of any commercial or financial relationships that could be construed as a potential conflict of interest.

## Publisher’s Note

All claims expressed in this article are solely those of the authors and do not necessarily represent those of their affiliated organizations, or those of the publisher, the editors and the reviewers. Any product that may be evaluated in this article, or claim that may be made by its manufacturer, is not guaranteed or endorsed by the publisher.

## Footnotes

^1^To generate these values, the latent path model was re-estimated using a maximum likelihood estimator.
