# Supplementary material for: Thriving Through Relationships in Sport: The Role of the Parent–Athlete and Coach–Athlete Attachment Relationship
Source: Front Psychol. 2021 Aug 2;12:694599. doi: 10.3389/fpsyg.2021.694599 (PMC8366224; doi:10.3389/fpsyg.2021.694599)
Supplement: Supplementary file 1 [file Data_Sheet_1.pdf]

Title: Thriving through relationships in sport: The role of the parent-athlete and coach-athlete attachment relationship

Journal: Frontiers in Psychology

Authors: Louise Davis, Daniel J. Brown, Rachel Arnold, and Henrik Gustafsson

Corresponding Author's Affiliation: Umeå University, Sweden

Corresponding Author's E-mail Address: louise.davis@umu.se

Electronic Supplementary Material: Example Mplus Syntax

The following supplementary material provide examples of the Mplus input files used for the analyses. These are intended to support readers' interpretation of the analyses conducted in the study and to offer substantive examples for their future work.

Example 1: Measurement Model

```
TITLE:
AT, NS, and TH Measurement Model
DATA:
File is "N:\Desktop\AT1.7.csv";
VARIABLE:
   NAMES ARE
   UniqueID
   CAAS1AV1 CAAS2AV2 CAAS3AV3 CAAS4AV4 CAAS5AV5 CAAS6AV6
CAAS7AV7
   CAAS8AX1 CAAS9AX2 CAAS10AX3 CAAS11AX4 CAAS12AX5 CAAS13AX6
CAAS14AX7
   CAAS15S1 CAAS16S2 CAAS17S3 CAAS18S4 CAAS19S5
   AS CS RS
   SP SV PA;
   USEVARIABLES
   CAAS1AV1 CAAS2AV2 CAAS3AV3 CAAS4AV4 CAAS5AV5 CAAS6AV6
CAAS7AV7
   CAAS8AX1 CAAS9AX2 CAAS10AX3 CAAS11AX4 CAAS12AX5 CAAS13AX6
CAAS14AX7
   CAAS15S1 CAAS16S2 CAAS17S3 CAAS18S4 CAAS19S5
   AS CS RS
   SP SV PA;
   MISSING = ALL(9999);
ANALYSIS:
   ESTIMATOR = MLR;
MODEL:
AVOID by CAAS1AV1 CAAS2AV2 CAAS3AV3 CAAS4AV4 CAAS5AV5 CAAS6AV6
CAAS7AV7;
      [AVOID];
      [CAAS1AV1@0];

ANX by CAAS8AX1 CAAS9AX2 CAAS10AX3 CAAS11AX4 CAAS12AX5 CAAS13AX6
CAAS14AX7;
      [ANX];
      [CAAS8AX1@0];

SECUR by CAAS15S1 CAAS16S2 CAAS17S3 CAAS18S4 CAAS19S5;
      [SECUR];
      [CAAS15S1@0];

NS by AS CS RS;
    [NS];
```

```
    [AS@0];

TH by SP SV PA;
     [TH];
     [SP@0];

OUTPUT:
   STDYX;
   MODINDICES(ALL);
   Cinterval;
```

Example 2: Direct Paths

```
TITLE:
AT and TH Direct Model
DATA:
File is "N:\Desktop\AT1.7.csv";
VARIABLE:
   NAMES ARE
   UniqueID
   CAAS1AV1 CAAS2AV2 CAAS3AV3 CAAS4AV4 CAAS5AV5 CAAS6AV6
CAAS7AV7
   CAAS8AX1 CAAS9AX2 CAAS10AX3 CAAS11AX4 CAAS12AX5 CAAS13AX6
CAAS14AX7
   CAAS15S1 CAAS16S2 CAAS17S3 CAAS18S4 CAAS19S5
   AS CS RS
   SP SV PA;
   USEVARIABLES
   CAAS1AV1 CAAS2AV2 CAAS3AV3 CAAS4AV4 CAAS5AV5 CAAS6AV6
CAAS7AV7
   CAAS8AX1 CAAS9AX2 CAAS10AX3 CAAS11AX4 CAAS12AX5 CAAS13AX6
CAAS14AX7
   CAAS15S1 CAAS16S2 CAAS17S3 CAAS18S4 CAAS19S5
   SP SV PA;
   MISSING = ALL(9999);
ANALYSIS:
   ESTIMATOR = MLR;
MODEL:
AVOID by CAAS1AV1 CAAS2AV2 CAAS3AV3 CAAS4AV4 CAAS5AV5 CAAS6AV6
CAAS7AV7;
      [AVOID];
      [CAAS1AV1@0];

ANX by CAAS8AX1 CAAS9AX2 CAAS10AX3 CAAS11AX4 CAAS12AX5 CAAS13AX6
CAAS14AX7;
      [ANX];
      [CAAS8AX1@0];

SECUR by CAAS15S1 CAAS16S2 CAAS17S3 CAAS18S4 CAAS19S5;
      [SECUR];
```

```
        [CAAS15S1@0];

TH by SP SV PA;
      [TH];
      [SP@0];

TH on AVOID ANX SECUR;

OUTPUT:
   STDYX;
   MODINDICES(ALL);
   Cinterval;
```

Example 3: Mediation Model

```
TITLE:
AT, NS, and TH Path Model
DATA:
File is "N:\Desktop\AT1.7.csv";
VARIABLE:
   NAMES ARE
   UniqueID
   CAAS1AV1 CAAS2AV2 CAAS3AV3 CAAS4AV4 CAAS5AV5 CAAS6AV6
CAAS7AV7
   CAAS8AX1 CAAS9AX2 CAAS10AX3 CAAS11AX4 CAAS12AX5 CAAS13AX6
CAAS14AX7
   CAAS15S1 CAAS16S2 CAAS17S3 CAAS18S4 CAAS19S5
   AS CS RS
   SP SV PA;
   USEVARIABLES
   CAAS1AV1 CAAS2AV2 CAAS3AV3 CAAS4AV4 CAAS5AV5 CAAS6AV6
CAAS7AV7
   CAAS8AX1 CAAS9AX2 CAAS10AX3 CAAS11AX4 CAAS12AX5 CAAS13AX6
CAAS14AX7
   CAAS15S1 CAAS16S2 CAAS17S3 CAAS18S4 CAAS19S5
   AS CS RS
   SP SV PA;
   MISSING = ALL(9999);
ANALYSIS:
   ESTIMATOR = MLR;
MODEL:
AVOID by CAAS1AV1 CAAS2AV2 CAAS3AV3 CAAS4AV4 CAAS5AV5 CAAS6AV6
CAAS7AV7;
        [AVOID];
        [CAAS1AV1@0];

ANX by CAAS8AX1 CAAS9AX2 CAAS10AX3 CAAS11AX4 CAAS12AX5 CAAS13AX6
CAAS14AX7;
        [ANX];
        [CAAS8AX1@0];
```

```
SECUR by CAAS15S1 CAAS16S2 CAAS17S3 CAAS18S4 CAAS19S5;
        [SECUR];
        [CAAS15S1@0];

NS by AS CS RS;
      [NS];
      [AS@0];

TH by SP SV PA;
      [TH];
      [SP@0];

TH on AVOID ANX SECUR NS;
NS on AVOID ANX SECUR;

model indirect: TH ind AVOID;
model indirect: TH ind ANX;
model indirect: TH ind SECUR;

OUTPUT:
   STDYX;
   Cinterval;
```

Example 4: Indirect Path Confidence Intervals

```
TITLE:
AT, NS, and TH Path Model Indirect CIs
DATA:
File is "N:\Desktop\AT1.7.csv";
VARIABLE:
   NAMES ARE
   UniqueID
   CAAS1AV1 CAAS2AV2 CAAS3AV3 CAAS4AV4 CAAS5AV5 CAAS6AV6
CAAS7AV7
   CAAS8AX1 CAAS9AX2 CAAS10AX3 CAAS11AX4 CAAS12AX5 CAAS13AX6
CAAS14AX7
   CAAS15S1 CAAS16S2 CAAS17S3 CAAS18S4 CAAS19S5
   AS CS RS
   SP SV PA;
   USEVARIABLES
   CAAS1AV1 CAAS2AV2 CAAS3AV3 CAAS4AV4 CAAS5AV5 CAAS6AV6
CAAS7AV7
   CAAS8AX1 CAAS9AX2 CAAS10AX3 CAAS11AX4 CAAS12AX5 CAAS13AX6
CAAS14AX7
   CAAS15S1 CAAS16S2 CAAS17S3 CAAS18S4 CAAS19S5
   AS CS RS
   SP SV PA;
   MISSING = ALL(9999);
ANALYSIS:
```

```
   Bootstrap = 10000;
MODEL:
AVOID by CAAS1AV1 CAAS2AV2 CAAS3AV3 CAAS4AV4 CAAS5AV5 CAAS6AV6
CAAS7AV7;
        [AVOID];
        [CAAS1AV1@0];

ANX by CAAS8AX1 CAAS9AX2 CAAS10AX3 CAAS11AX4 CAAS12AX5 CAAS13AX6
CAAS14AX7;
        [ANX];
        [CAAS8AX1@0];

SECUR by CAAS15S1 CAAS16S2 CAAS17S3 CAAS18S4 CAAS19S5;
        [SECUR];
        [CAAS15S1@0];

NS by AS CS RS;
      [NS];
      [AS@0];

TH by SP SV PA;
      [TH];
      [SP@0];

TH on AVOID ANX SECUR NS;
NS on AVOID ANX SECUR;

model indirect: TH ind AVOID;
model indirect: TH ind ANX;
model indirect: TH ind SECUR;

OUTPUT:
   STDYX;
   Cinterval (bcbootstrap);
```

Example 5: Path Analysis for Parent Attachment, Performance, and Thriving

```
TITLE:
Father attachment, Performance, and TH(FScores) Structural Model
DATA:
File is "N:\Desktop\AT3.5.csv";
VARIABLE:
   NAMES ARE
      Number
      FSA
      FIA
      PER
      TH;
   USEVARIABLES
      FSA
```

```
      FIA
      PER
      TH;
   MISSING = ALL(9999);

ANALYSIS:
   ESTIMATOR = MLR;
MODEL:
TH ON FSA FIA;
PER ON FSA FIA;

OUTPUT:
   sampstat;
   STDYX;
   Cinterval;
```
